# Cellular and extracellular white matter alterations after childhood trauma experience in individuals with schizophrenia

**DOI:** 10.1017/S0033291724003064

**Published:** 2024-12

**Authors:** Maria R. Dauvermann, Laura Costello, Giulia Tronchin, Emma Corley, Laurena Holleran, David Mothersill, Karolina I. Rokita, Ruán Kane, Brian Hallahan, Colm McDonald, Ofer Pasternak, Gary Donohoe, Dara M. Cannon

**Affiliations:** 1 Center for Neuroimaging, Cognition and Genomics (NICOG), Clinical Neuroimaging Laboratory, Galway Neuroscience Centre, University of Galway, Galway, Ireland; 2 Institute for Mental Health, School of Psychology, University of Birmingham, Birmingham, UK; 3 Department of Psychology, School of Business, National College of Ireland, Dublin, Ireland; 4 Department of Psychiatry, Trinity College Dublin, St. James's Hospital, Dublin, Ireland; 5 Department of Radiology, Brigham and Women's Hospital, Harvard Medical School, Boston, MA, USA; 6 Department of Psychiatry, Brigham and Women's Hospital, Harvard Medical School, Boston, MA, USA; 7 Department of Psychiatry, Massachusetts General Hospital, Harvard Medical School, Boston, MA, USA

**Keywords:** childhood Trauma, diffusion-weighted magnetic resonance imaging, fractional anisotropy, free water, schizophrenia, tract-based spatial statistic

## Abstract

**Background:**

Childhood trauma (CT) is related to altered fractional anisotropy (FA) in individuals with schizophrenia (SZ). However, it remains unclear whether CT may influence specific cellular or extracellular compartments of FA in SZ with CT experience. We extended our previous study on FA in SZ (Costello et al., 2023) and examined the impact of CT on hypothesized lower free water-corrected FA (FA_T_) and higher extracellular free water (FW).

**Method:**

Thirty-seven SZ and 129 healthy controls (HC) were grouped into the ‘none/low’ or ‘high’ CT group. All participants underwent diffusion-weighted magnetic resonance imaging. We performed tract-based spatial statistics to study the main effects of diagnostic group and CT, and the interaction between CT and diagnostic group across FA_T_ and FW.

**Results:**

SZ displayed lower FA_T_ within the corpus callosum and corona radiata compared to HC (*p* < 0.05, Threshold-Free Cluster Enhancement (TFCE)). Independent of diagnosis, we observed lower FA_T_ (*p* < 0.05, TFCE) and higher FW (*p* < 0.05, TFCE) in both SZ and HC with high CT levels compared to SZ and HC with none or low CT levels. Furthermore, we did not identify an interaction between CT and diagnostic group (*p* > 0.05, TFCE).

**Conclusions:**

These novel findings suggest that the impact of CT on lower FA_T_ may reflect cellular rather than extracellular alterations in established schizophrenia. This highlights the impact of CT on white matter microstructure, regardless of diagnostic status.

## Introduction

It is widely known that the experience of childhood trauma (CT) is related to an increased likelihood of developing psychotic disorder (Quidé, Tozzi, Corcoran, Cannon, & Dauvermann, 2020; Trauelsen et al., 2015) and greater severity of symptoms in individuals with schizophrenia (SZ) (Bailey et al., 2018; Bendall, Jackson, Hulbert, & McGorry, 2007; Kelleher et al., 2013; Morgan & Fisher, 2006; Popovic et al., 2019; Varese et al., 2012). Furthermore, studies have shown that the experience of CT is associated with both structural and functional brain changes in SZ when compared to healthy controls (HC) (Cancel, Dallel, Zine, El-Hage, & Fakra, 2019; Costello et al., 2023; Dauvermann et al., 2021; Rokita et al., 2020). These changes may be related to abnormal white matter (WM) development that occurs as a consequence of early trauma (Heim & Nemeroff, 1999; McLaughlin, DeCross, Jovanovic, & Tottenham, 2019). However, some of these fractional anisotropy (FA) findings in SZ have been inconsistent. In this study, we extended our previous approach (Costello et al., 2023) to further examine whether lower FA, particularly in deep white matter voxels encompassing frontolimbic and frontoparietal regions, is due to cellular alterations or confounded by contributions from extracellular components.

Inconsistencies in findings may be amplified by the use of conventional diffusion-based FA metrics, which lack specificity to microstructural changes and often reflect a combination of cellular and extracellular components, thereby limiting the interpretation of results (Pasternak et al., 2009). While not specific, the FA measure is sensitive to biophysical properties of WM, such as fiber orientation, axonal diameter, packing density, myelination, and membrane permeability. Partial volume effects (PVE), i.e., mixed tissue types in a WM voxel may also occur (Basser & Pierpaoli, 2011; Beaulieu, 2002; Beaulieu & Allen, 1994). In the case of PVE, indices of FA reflect a mixture of both cellular and extracellular components, which limits the interpretation of biological specificity of the findings (Albi et al., 2017; O'Donnell & Pasternak, 2015). Partial volume contamination with free water (FW) is particularly problematic in WM regions adjacent to fluid-filled spaces and at the boundaries of the brain parenchyma, such as the cingulum, the fornix, and parts of the corpus callosum. Therefore, it is possible that some of the previously documented microstructural reductions within these regions are attributable to extracellular changes (Alexander, Hasan, Lazar, Tsuruda, & Parker, 2001; Bergamino, Walsh, & Stokes, 2021; Cetin-Karayumak et al., 2020; Ellison-Wright & Bullmore, 2009; Fitzsimmons, Kubicki, & Shenton, 2013; Metzler-Baddeley, O'Sullivan, Bells, Pasternak, & Jones, 2012; Pasternak, Kelly, Sydnor, & Shenton, 2018; Samartzis, Dima, Fusar-Poli, & Kyriakopoulos, 2014).

One promising approach to remove the PVE of FW in tissue is the bi-tensor free water elimination (FWE) model (Pasternak et al., 2009). This FWE model decompartmentalizes the diffusion signal into two biological components. The first component reflects the contribution of freely diffusing water molecules in the extracellular space known as the fractional volume of FW. The second component represents the signal that is left in the tissue following the elimination of extracellular FW, which can be fitted to a diffusion-tensor (Pasternak et al., 2009). At the cellular level, free water-corrected FA (FA_T_) measures are thought to be more sensitive to anatomical changes than standard DTI-derived indices (Bergamino et al., 2021; Metzler-Baddeley et al., 2012). In contrast, at the extracellular space, elevated FW may reflect processes, such as atrophy, oedema, and neuroinflammation (Di Biase et al., 2021; Pasternak, Kubicki, & Shenton, 2016).

The FWE model has been applied to diffusion data in SZ (Pasternak et al., 2018; Wu et al., 2024) and separately in HC with CT experience (McCarthy-Jones et al., 2018). In SZ, both higher and lower FA_T_ were reported in SZ when compared to HC, particularly in the corpus callosum, posterior thalamic radiation, corona radiata, and internal capsule (Oestreich et al., 2017; Pasternak, Westin, Dahlben, Bouix, & Kubicki, 2015; Wu et al., 2024). These microstructural changes of lower FA_T_ at a cellular level suggest that some of the existing findings of widespread microstructural differences may have been biased by the influence of isotropic components (Bergamino, Pasternak, Farmer, Shenton, & Paul Hamilton, 2016; Metzler-Baddeley et al., 2012). Additionally, the isolation of both cellular and extracellular findings within a voxel can be summarized with higher extracellular FW that appear to be more prominent in the earlier stages of the disorder (Bergé et al., 2020; Cetin-Karayumak et al., 2020; Guo et al., 2020; Lesh et al., 2021; Lyall et al., 2018; Pasternak et al., 2018). Comparable to the findings in schizophrenia, when using FA_T_ in HC who have a history of CT, McCarthy-Jones et al. (2018) found lower FA_T_ in the corona radiata, corpus callosum, and uncinate fasciculus. However, no differences in FW were observed, which may be linked to the chosen region of interest (ROI) approach (McCarthy-Jones et al., 2018). Despite these findings demonstrating a regional impact of CT and SZ independently on cellular and extracellular components, no study to date has examined the influence of CT in SZ.

The current study extends our previous findings in the same participants (Costello et al., 2023) and aims to investigate the impact of CT in SZ on cellular (FA_T_) and extracellular components in WM tissue (FW) by using FWE (Pasternak et al., 2009). Our first hypothesis was that SZ would display lower FA_T_ in localized regions of the brain such as the corona radiata and corpus callosum based on earlier studies (Costello et al., 2023; McCarthy-Jones et al., 2018) compared to HC. Secondly, we assumed that both SZ and HC with CT would show lower FA_T_ and higher in FW, specifically in deep WM voxels encompassing fronto-limbic and fronto-parietal regions, compared with SZ and HC without CT. Finally, we predicted that lower FA_T_ and higher FW would be associated in SZ with high CT severity levels, whereas this relationship would not be observed in HC with high CT levels and participants with none or low CT levels.

## Methods

This study is an extension of our previous approach of examining the impact of CT on FA (Costello et al., 2023). Participants were recruited as part of the ‘Immune Response & Social Cognition in Schizophrenia’ project (iRELATE) and the ‘Social cognition study in schizophrenia’ with identical eligibility criteria, questionnaires, assessments, and MRI brain acquisition parameters. Details on the recruitment process have been previously reported (Costello et al., 2023; Dauvermann et al., 2021). Inclusion criteria required participants to be aged between 18 and 65 years of age and of Irish or British origin. SZ were included in the study if they had a confirmed diagnosis of schizophrenia/schizoaffective disorder assessed using the Structured Clinical Interview (SCID) for Diagnostic and Statistical Manual of Mental Disorders (DSM-IV-TR) by a trained clinical psychiatrist. Exclusion criteria for all participants included documented evidence of a head injury resulting in loss of consciousness more than one minute (based on self-report), intellectual disability (IQ < 70), neurological disorder, pregnant/breastfeeding, alcohol or substance use disorder, and contraindication to MRI scanning. HC with a self-reported history of mental health problems, antipsychotic medication use, or a first-degree relative with SZ disorder were excluded from participating.

Clinical symptom severity was assessed using the Positive and Negative Syndrome Scale (PANSS); (Kay, Fiszbein, & Opler, 1987). PANSS items were rated on a seven-point scale with items on each subscale ranging from ‘1’ (‘absent’) to ‘7’ (‘extreme’) consistent with previous publications from the iRELATE study (Costello et al., 2023; Dauvermann et al., 2021). We used the re-scaled Likert scale ranging from ‘0’ (‘absent’) to ‘6’ (‘extreme’) resulting in a total score ranging from ‘0’ to ‘138’ to identify ‘absence’ scores (Leucht et al., 2015). Antipsychotic treatment was recorded and a chlorpromazine equivalent dose (CPZ) was calculated as described in detail previously (Andreasen, Pressler, Nopoulos, Miller, & Ho, 2010; Leucht et al., 2015) and keeping with (Costello et al., 2023; Dauvermann et al., 2021).

CT was assessed with the Childhood Trauma Questionnaire (CTQ) (Bernstein et al., 2003). Briefly, the CTQ is the most widely established retrospective self-reported questionnaire to assess five subtypes of abuse (emotional, physical, and sexual) and/or neglect (emotional and physical) that may have occurred during childhood and adolescence until the age of 18 years (Bernstein et al., 2003). The items on each subscale range from ‘1’ (‘never true’) to ‘5’ (very often true). In line with our previous approach to highlight ‘absence’ scores for iRELATE publications (Costello et al., 2023; Dauvermann et al., 2021), the CTQ scores were rescaled ranging from ‘0’ (‘never true’) to ‘4’ (‘very often true’). Consequently, rescaled scores resulted in total scores ranging from ‘0 to 100’ instead of ’25 to 125’.

As previously reported in detail (Costello et al., 2023), all SZ and HC were allocated into the groups of either ‘high levels of trauma’ or ‘none-low levels of trauma’ following our previous approach. For the analysis, the CTQ dichotomy was coded as 1 (no trauma) and 2 (trauma). High levels denote the occurrence of moderate or severe abuse, whereas none or low levels resemble CT events that never happened or where a single event of moderate or severe neglect was reported (Table 1) (Please note that Table 1 and Supplemental Tables 2 and 3 are replicated (Costello et al., 2023).). Details on the original and rescaled total CTQ and subtype scores in all participants are presented in the supplemental material. Furthermore, we also provided details on the CT severity across each of the five CTQ subtypes (Supplemental Table 2)^.^

**Table 1.** Demographic and clinical characteristics of all participants stratified by study group and trauma severity (*n* = 166)

Demographic and clinical characteristics	Diagnostic groups				Severity categorisation of trauma severity	
	Healthy controls	Individuals with schizophrenia	Statistical comparison between groups (*U*/χ^2^, *p* value)	None/low	High	Statistical comparison between groups (*U*/χ^2^, *p* value)
Sample size, *n*	129 (78%)	37 (22%)			118 (71%)	48 (29%)
Age (years), mean ± s.d.	34 ± 11	44.0 ± 11	*U* = 1248.5, *p* < 0.001	35 ± 11	39 ± 14	*U* = 2379.5, *p* = 0.11
Sex, female (*n*, %)/males (*n*, %)	70 (54.3%)/59 (45.7%)	26 (70.3%)/11 (29.7%)	χ^2^ = 3.02, *p* = 0.08	73 (61.9%)/45 (38.1%)	23 (47.9%)/25 (52.1%)	χ*^2^* = 2.72, *p* = 0.10
Handedness, right (*n*, %)/left (*n*, %)	122 (94.6%)/7 (5.4%)	35 (94.6%)/2 (5.4%)	χ^2^ = 0.58, *p* = 0.75	111 (94.1%)/7 (5.9%)	46 (95.8%)/2(4.2%)	χ^2^ = 1.05, *p* = 0.59
Years of education (years), mean ± s.d.	16.8 ± 4.1	13.8 ± 4.4	*U* = 977.5, *p* = 0.00003	16.0 ± 4.3	16.8 ± 4.3	*U* = 2065.0, *p* = 0.18
CTQ total (0–100), mean ± s.d.	11.53 ± 12.02	18.05 ± 14.6	*U* = 1516.0, *p* = 0.001	7.07 ± 5.78	27.5 ± 14.0	*U* = 402.0, *p* = 4.52 × 10^−18^
Emotional abuse (0–20), mean ± s.d.	3.47 ± 4.20	5.22 ± 4.81	*U* = 1778.0, *p* *=* 0.02	2.0 ± 2.04	8.44 ± 5.21	*U* = 718.0, *p* *=* 2.77 × 10^−14^
Physical abuse (0–20), mean ± s.d.	1.43 ± 2.29	2.81 ± 4.37	*U* = 1962.0, *p* *=* 0.08	0.73 ± 1.16	4.23 ± 4.22	*U* = 1086.0, *p* = 2.96 × 10^−11^
Sexual abuse (0–20), mean ± s.d.	0.96 ± 2.95	1.70 ± 4.44	*U* = 2319.5, *p* *=* 0.69	0.09 ± 0.43	3.69 ± 5.41	*U* = 1557.5, *p* *=* 6.85 × 10^−12^
Emotional neglect (0–20), mean ± s.d.	1.54 ± 2.64	3.08 ± 3.39	*U* = 1609.5, *p* = 0.002	3.46 ± 3.58	7.35 ± 5.02	*U* = 1459.0, *p* = 8.46 × 10^−7^
Physical neglect (0–20), mean ± s.d.	0.63 ± 0.94	0.51 ± 0.93	*U* = 1483.0, *p* *=* 0.002	1.08 ± 1.74	3.85 ± 4.02	*U* = 1552.5, *p* *=* 1.0 × 10^−6^
HAM-D (0–17), mean ± s.d.	3.0 ± 3.61	4.06 ± 4.26	*U* = 1262.5, *p* = 0.20	4.25 ± 4.58	3.73 ± 3.80	*U* = 1505.5, *p* = 0.32
Duration of illness (years)	–	19 ± 11	–	16 ± 9.10	23 ± 12.70	*U* = 59.0, *p* = 0.15
PANSS, positive (7–49), mean ± s.d.	–	8.44 ± 2.20	–	7.85 ± 1.53	9.64 ± 2.8	*U* = 86.0, *p* = 0.25
PANSS, negative (7–49), mean ± s.d.	–	9.84 ± 4.10	–	9.85 ± 4.15	10.09 ± 4.21	*U* = 106.0, *p* = 0.73
PANSS, total (30–210), mean ± s.d.	–	38.78 ± 9.16	–	37.75 ± 7.38	40.8 ± 12.24	*U* = 103.5, *p* = 0.64
CPZ equivalents, mean ± s.d.	–	517.3 ± 357.9	–	438.6 ± 326.1	643.4 ± 387.1	*U* = 52.0, p = 0.15
	Categorisation of trauma severity across study groups					
	Healthy control: none/low	Healthy control: high		Schizophrenia: none/low	Schizophrenia: high	Statistical comparison between groups (*H/*χ^2^, *p* value)
Sample size, *n*	94	35		24	13	
Age (years), mean ± s.d.	33 ± 11	36 ± 13	–	42 ± 9	47 ± 14	*H*(*3*) = 21.18, *p* = 0.00009
Sex, males/female (*n*, %)	55 (58.5%)/39 (41.5%)	15 (42.9%)/20 (57.1%)	–	18 (75.0%)/6 (25.0%)	8 (61.5%)/5 (38.4%)	χ^2^ = 6.21, *p* = 0.10
Handedness, right/left (*n*, %)	89 (94.6%)/5 (5.4%)	33 (94.3%)/2 (5.7%)	–	22 (91.7%)/2 (8.3%)	13 (100%)/0 (0%)	χ^2^ = 2.70, *p* = 0.84
Years of education (years), mean ± s.d.	16.4 ± 4.4	17.9 ± 3.17	–	14.1 ± 3.8	13.1 ± 5.5	*H*(*3*) = 20.4, *p* = 0.0001
CTQ total-RS (0–100), mean ± s.d.	6.18 ± 5.5	25.89 ± 13.12	–	10.54 ± 5.83	31.9 ± 15.83	*H*(*3*) = 82.55, *p* = 8.72 × 10^−18^
Emotional abuse (0–20), mean ± s.d.	1.73 ± 1.84	8.14 ± 5.12	–	3.04 ± 2.44	9.23 ± 5.58	*H* = 62.11, *p* *=* 2.08 × 10^−13^
Physical abuse (0–20), mean ± s.d.	0.67 ± 1.08	3.49 ± 3.25	–	0.96 ± 1.43	6.23 ± 5.82	*H* = 46.52, *p* *=* 4.39 × 10^−10^
Sexual Abuse (0–20), Mean ± s.d.	0.09 ± 0.44	3.31 ± 4.94	–	0.08 ± 0.41	4.69 ± 6.63	*H* = 47.10, *p* *=* 3.33 × 10^−10^
Emotional neglect (0–20),Mean ± s.d.	2.81 ± 3.08	7.54 ± 5.13	–	6.0 ± 4.30	6.85 ± 4.86	*H* = 35.69, *p* = 8.71 × 10^−8^
Physical neglect (0–20), Mean ± s.d.	0.85 ± 4	3.40 ± 3.87	–	2.0 ± 2.17	5.08 ± 4.33	*H* = 35.36, *p* *=* 1.02 × 10^−7^

CTQ, Childhood Trauma Questionnaire; HC, healthy controls; S.D., standard deviation; SZ, individuals with schizophrenia.

Demographic and clinical characteristics presented for all participants and stratified according to either study groups (HC or SZ), severity level of trauma independent of diagnosis (high levels of trauma or none/low levels of trauma) resulting in the following groups: (i) HC; (ii) SZ; (iii) none/low levels of trauma; (iv) high level of trauma; (v) HC with none/low levels of trauma; (vi) HC with high levels of trauma; (vii) SZ none/low levels of trauma; (viii) SZ high levels of trauma.

### Magnetic resonance imaging data acquisition and processing

Diffusion-weighted MR images (DWI) were acquired for all participants at the Wellcome Trust Health Research Board National Centre for Advanced Medical Imaging (CAMI) at St. James's Hospital Dublin, Ireland, using a 3.0 Tesla Achieva scanner with a 32-direction Stejskal–Tanner diffusion encoding scheme (Philips Medical Systems, Best, The Netherlands). DWI data acquisition and basic processing details have been described in detail previously (Costello et al., 2023) and are presented in the Supplemental Material.

#### Free water elimination model

FA_T_ and FW maps were computed for all participants by fitting a FWE model in each voxel of pre-processed diffusion-weighted images using an in-house script (MATLAB v7.1); (Pasternak et al., 2009). In the FWE model, the diffusion coefficient of free water is set to the diffusivity of water at body temperature (3 × 10^−3^ mm^2^/s) so that the isotropic diffusion of water molecules in the extracellular space can be modeled within each voxel to obtain extracellular FW maps (Pasternak et al., 2009). The tissue component of the FWE model represents the diffusion signal left in the cellular tissue following the elimination of extracellular free water (Pasternak et al., 2009). This signal was fitted to a diffusion tensor and converted to a scalar metric using the diffusion-tensor eigenvalue decomposition to obtain a free-water corrected measure of FA, otherwise known as tissue-specific anisotropy (FA_T_).

#### Statistical analyses

Tract-based spatial statistics (TBSS) was used to assess group differences in FA_T_ and FW across deep WM voxels (FSL v6.0.1); (Smith et al., 2006). SZ and HC were compared across demographic, clinical, and environmental measures (e.g. age, sex, handedness, and years of education) using Mann–Whitney *U* test or chi-square tests and a significance threshold *p* < 0.05 (SPSS v25).

We ran multivariate analysis of covariance (MANCOVA) separately for FA_T_ and FW parameters covarying for age and sex. We performed a single model to test for two main effects with two levels (i.e. main effect of diagnostic group and main effect of CT) on FA_T_ and FW and the interaction between diagnostic group and CT.

We ran voxel-wise statistics using permutation-based analysis (*n* = 5000) (Winkler, Ridgway, Webster, Smith, & Nichols, 2014). Measures of FA_T_ and FW were extracted from significant clusters of masked voxels using the *fslmeants* cluster tool (FSL v6.0.1; (Jenkinson, Beckmann, Behrens, Woolrich, & Smith, 2012). A ‘cluster’ denoted a significant region(s) of the brain identified in our analysis between groups.

Pearson's partial correlation assessed the relationships between FA_T_ extracted from significant clusters and clinical variables, including symptom severity as measured using PANSS scores, duration of illness, and CPZ in SZ.

## Results

SZ were significantly older than HC (*p* < 0.001), while there were no group differences for sex and handedness. All SZ were treated with at least one type of atypical antipsychotic medication.

The distribution of self-reported CT severity has been reported in detail previously (Costello et al., 2023). Briefly, 48 participants were allocated to the ‘high levels’ group (HC = 35, SZ = 13, Table 1), while 118 participants were assigned to the ‘none or low levels’ group (HC = 94, SZ = 24, Table 1). Our previous non-parametric Mann–Whitney *U* test showed no significant differences for age or sex between the none/low and moderate/high CT severity groups. However, we observed a significant difference for the total CTQ score between none/low and moderate/high CT levels (*p* < 0.05, Table 1). CT severity levels across SZ and HC were comparable for each of the five CTQ subscales and the groups did not differ in relation to sex, handedness, or clinical variables (*p* > 0.05, Table 1, (Costello et al., 2023)).

**Figure 1.**
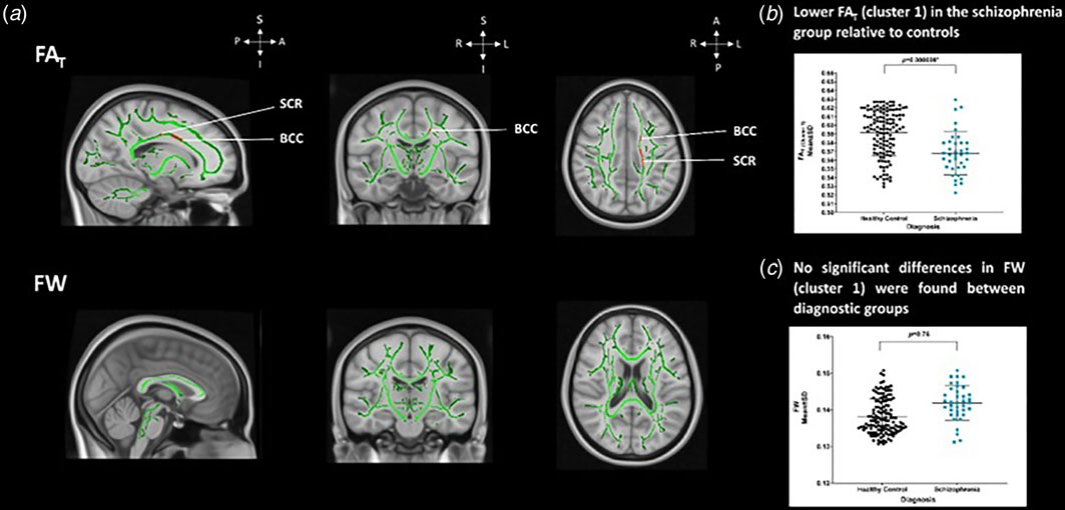
Free water-corrected fractional anisotropy and extracellular free water. (a) Top. Significant differences in FA_T_ between SZ than HC in a cluster encompassing the BCC, left SCR and PCR (*p* < 0.05*, TFCE). Significance denoted in a red (*p* = 0.05) to yellow (lowest *p* value) colour intensity scale (*x* = 90, *y* = 108, *z* = 90). Bottom. No significant differences in extracellular FW between SZ than HC. (b) Scatterplot shows extracted FA_T_ values extracted from significant cluster (a) of lower FA_T_ (*p* < 0.05*, TFCE) and plotted across diagnostic groups of SZ and HC. (c) Scatterplot illustrates the non-significant differences in FW using values extracted from the group-based FW template and plotted across diagnostic groups for SZ and HC for visualisation purposes (*p* > 0.05, TFCE). *p* values in the graph demonstrate the post-hoc results of group differences in the extracted FA_T_ (B, Cluster, *p* < 0.05*, MANCOVA) and FW values (C, *p* > 0.05, MANCOVA). Error bars represent mean and standard deviation and the 95% confidence intervals for all diagnostic groups, and an asterisk denotes statistically significant differences between groups (*p* < 0.05*). BCC, body of the corpus callosum; FA_T_, free water-corrected fractional anisotropy; FW, extracellular free water; HC, healthy controls; PCR, posterior corona radiata; SCR, superior corona radiata, SZ, individuals with schizophrenia.

We observed two significant clusters of lower FA_T_ in SZ relative to HC, which encompassed the body of corpus callosum, and the left superior (Cluster 1) and posterior corona radiata (Cluster 2) (*p* < 0.05, TFCE; Figure 1a and 1b, Supplemental Table 3). We did not find any significant differences in FW between SZ and HC (*p* > 0.05, TFCE; Figure 1a and 1c). A post-hoc correlation revealed that variances in mean FA_T_ across the significant cluster did not relate to differences in clinical measures: PANSS positive (*r* = −0.34, *p* = 0.06), PANSS negative (*r* = 0.04, *p* = 0.84), PANSS total scores (*r* = −0.95, *p* = 0.61), illness duration (*r* = −0.19, *p* = 0.39), or CPZ scores (*r* = 0.05, *p* = 0.82).

**Figure 2.**
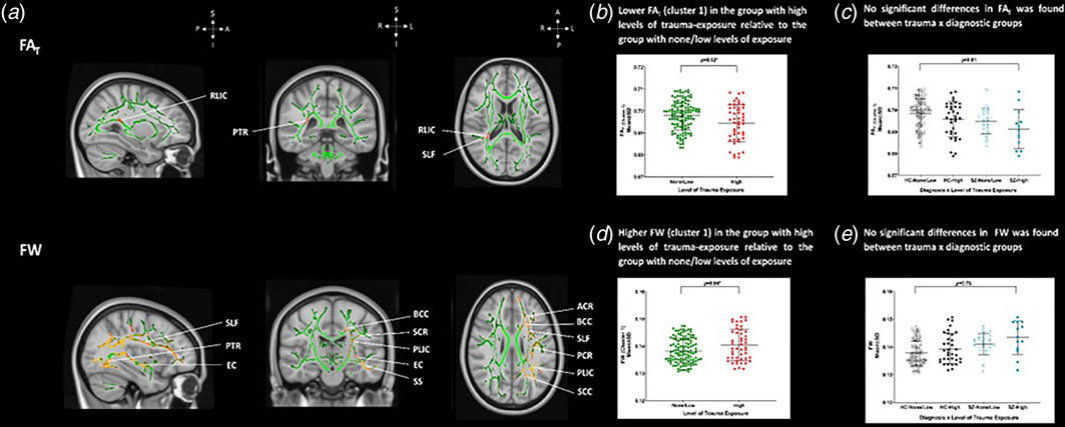
CT and lower free water-corrected fractional anisotropy in frontolimbic regions. (a) Top. White matter voxels of significantly lower FA_T_ in SZ than HC. The significant white matter cluster of lower FA involved the posterior corona radiata, retrolenticular limb of the internal capsule, posterior thalamic radiation, and superior longitudinal fasciculus, *p* < 0.05, (TFCE). Bottom. Significantly higher FW was found in a large white matter cluster involving the corpus callosum, body of the corpus callosum, genu of the corpus callosum, splenium of the corpus callosum, the left corona radiata, anterior corona radiata, superior corona radiata and posterior corona radiata, the limb of the internal capsule, anterior limb of the internal capsule, posterior limb of the internal capsule, retrolenticular limb of the internal capsule, superior longitudinal fasciculus, external capsule, posterior thalamic radiation, cerebral peduncle, uncinate fasciculus, superior fronto-occipital fasciculus, tapetum, and the sagittal stratum, cluster, *p* < 0.05, (TFCE). Significant white matter voxels with lower FA_T_ and higher FW are denoted in a red (*p* < 0.05) to yellow (lowest *p* value) colour intensity scale and are overlaid on the subject-specific white matter skeleton, shown in green across all three orthogonal views (coronal, axial, and sagittal) displayed in radiological format (*X* = 90, *Y* = 108, *Z* = 90). (b) The scatterplot illustrates lower FA_T_ values in participants with CT (*p* < 0.05*, TFCE). (C) The scatterplot illustrates no significant differences in FA_T_ between SZ with high CT levels, HC with high CT levels, SZ with none/low CT levels and HC with none/low CT levels of exposure (*p* < 0.05*, TFCE). (c) The scatterplot illustrates higher FW in participants with CT (*p* < 0.05*, TFCE). (d) The scatterplot illustrates the non-significant FW clusters of higher FW were detected between CT groups (*p* > 0.05, TFCE). *p* values in the graph demonstrate the post-hoc results of group differences in the extracted FA_T_ (B, *p* < 0.05*, MANCOVA) and FW values (C, *p* > 0.05, MANCOVA). Error bars represent mean and standard deviation and the 95% confidence intervals for all groups, and an asterisk denotes statistically significant differences between groups (*p* < 0.05*). HC, healthy controls; SZ, individuals with schizophrenia.

We observed significantly lower FA_T_ across all participants of SZ and HC with CT experience compared with SZ and HC without CT history, in a cluster of deep WM voxels encompassing the right posterior corona radiata, retrolenticular part of the internal capsule, posterior thalamic radiation (optic radiation), and the superior longitudinal fasciculus (*p* < 0.05, TFCE; Fig. 2a, 2b, Table S4). We did not find statistically significant clusters of higher FA_t_ between the different groups listed in the tables (*p* > 0.05, TFCE), including comparisons of low *v.* high CT levels within HC and SZ. Furthermore, there was no significant interaction between CT severity levels and diagnostic group (*p* > 0.05, TFCE; Fig. 2c).

After covarying for age and sex, we did not find any significant differences in extracellular FW in SZ compared to HC (*p* > 0.05, TFCE; Fig. 1a). When testing for the main effect of CT we found significantly higher FW in participants with CT experience across both SZ and HC compared with SZ and HC without such CT history. We identified the most pronounced finding in a large cluster involving the genu, body, and splenium of the corpus callosum, fornix, the left anterior, superior, and posterior corona radiata, anterior, posterior and retrolenticular limb of the internal capsule, superior longitudinal fasciculus, external capsule, posterior thalamic radiation (optic radiation), tapetum, cerebral peduncle, uncinate fasciculus, sagittal stratum, and the superior fronto-occipital fasciculus (*p* < 0.05, TFCE; Fig. 2a, 2d, Table S5). We did not observe any significant interactions in FW between CT and diagnosis group (*p* > 0.05, TFCE; Fig. 2e).

We note that these analyses were performed using CTQ scores as a categorical variable. In a separate run, we reran these analyses with CTQ scores as a continuous variable, and while the general pattern of results remained, there were fewer significant associations. Specifically, for FA, the main regions showing effects included the forceps minor, uncinate fasciculus, anterior thalamic radiation, and inferior longitudinal fasciculus. For FW, the regions included the forceps minor and major, inferior longitudinal fasciculus, internal capsule, and corpus callosum (Supplementary Figs 1 and 2).

## Discussion

The aim of this study was to examine whether having SZ with exposure to CT was associated with lower FA_T_ and higher extracellular FW compared to HC. The main findings were that the SZ group displayed lower FA_T_ in the corpus callosum and corona radiata relative to HC. However, we did not observe an effect of SZ on FW when compared to HC. Furthermore, we found that self-reported CT affected both SZ and HC participants, showing associations with lower FA_T_ in the corona radiata, the internal capsule and superior longitudinal fasciculus and greater extracellular FW. We did not find an interaction between CT and diagnostic group for FA_T_ or FW.

The first main finding was that we observed lower FA_T_ in the body of the corpus callosum and the left superior and posterior corona radiata in SZ when compared to HC. These findings extend findings of lower FA in overlapping regions in SZ (Costello et al., 2023). Furthermore, we did not find any differences in extracellular FW between SZ and HC, in keeping with previous studies, which suggest the later stages of schizophrenia may be characterized by cellular rather than extracellular alterations (Oestreich et al., 2017; Pasternak et al., 2018, 2015). However, a recent study reported increased FW in commissural, association, and projection fibers in individuals with SZ (Wu et al., 2024), potentially due to older SZ with a longer duration of illness herein. We identified lower FA_T_ in the same regions of the corpus callosum and corona radiata as highlighted by prior DTI studies demonstrating the involvement of interhemispheric WM (Kelly et al., 2018; Rotarska-Jagiela et al., 2008; Whitford et al., 2010) and projection regions in SZ (Kelly et al., 2018; Kubicki, McCarley, & Shenton, 2005).

While our findings are largely in agreement with previous DWI studies in SZ, we note that we did not replicate findings of lower FA_T_ in either the superior longitudinal fasciculus bilaterally, left external capsule (Pasternak et al., 2015), anterior limb of the internal capsule, or the posterior thalamic radiation (Oestreich et al., 2017). Moreover, we also did not find any significant relationship between FA_T_ and clinical variables in SZ as previously reported (Pasternak et al., 2015). This discrepancy could reflect clinical differences since our findings were based on a modest sample size of individuals with established SZ, who presented with mild clinical symptoms at the time of assessment. Similarly, all individuals with SZ were taking antipsychotic medication at the time of scanning, which may have minimized groups findings due to a hypothesized promyelinating effect (Barth et al., 2020; Lawrie, 2018). Identifying the involvement of WM alterations in the pathophysiology of SZ, which are independent from further related effects is challenging in a cross-sectional study of individuals with a long duration of illness. Therefore, the potential confounding effects of illness duration, cumulative medication use, and symptom severity could not be adequately explored in this study. Furthermore, we note that the smaller extent of findings here compared to previous studies could also be related to the significant age difference between the SZ and HC groups.

We reported novel findings with both SZ and HC, who have experienced CT, showing lower FA_T_ in the corona radiata, the internal capsule, the superior longitudinal fasciculus and other interconnected regions when compared to SZ and HC without CT history. This cluster may appear extensive due to the use of the TFCE method, which tends to generate more spatially extensive clusters, especially in highly interconnected regions such as those identified in our analysis. The lower FA_T_ in our study replicated previously reported FA associations with CT in late-developing fronto-thalamic WM regions (Choi, Jeong, Polcari, Rohan, & Teicher, 2012; Huang, Gundapuneedi, & Rao, 2012; Lim et al., 2019; Teicher et al., 2016). Furthermore, our findings are partially in keeping with the only other available study that used FA_T_ in healthy participants who have experienced CT (McCarthy-Jones et al., 2018).

In contrast to McCarthy-Jones et al. (2018), we did not find a relationship between CT and lower FA_T_ in either the corpus callosum or the uncinate fasciculus. Possible explanations for this discrepancy may be due to the difference in studied population, where we included both SZ and HC in contrast to only HC with a history of CT (McCarthy-Jones et al., 2018). In addition, we used a whole-brain approach, whereas McCarthy-Jones et al., applied a ROI approach (McCarthy-Jones et al., 2018). Furthermore, it is possible that differences in age at which CT was experienced as well as possible differences in the severity and frequency of CT subtypes may have contributed towards the discrepancy in findings (Croft et al., 2019; Fisher et al., 2010). Finally, these findings need to be considered in the context of a potential resilience, compensation, or adaptation impact, as lower FA_T_ in the corona radiata, the internal capsule, and superior longitudinal fasciculus across SZ and HC in our study is comparable to reduced FA of the superior longitudinal fasciculus in individuals with CT history but without a diagnosis of a neuropsychiatric disorder, indicating potential resilience in the HC group (Teicher et al., 2016; Teicher, Ohashi, & Khan, 2020). Future longitudinal studies are required to examine the involvement of such processes.

Both SZ and HC with CT experience showed higher extracellular FW across the corpus callosum corona radiata, internal capsule, posterior thalamic radiation, superior longitudinal fasciculus, external capsule, and uncinate fasciculus potentially informing past studies reporting lower FA (Choi et al., 2012; DeRosse, Ikuta, Karlsgodt, Szeszko, & Malhotra, 2020; Hanson et al., 2013; Lim et al., 2019; Rinne-Albers, van der Wee, Lamers-Winkelman, & Vermeiren, 2013), as indicative of extracellular FW rather than tissue-related microstructural differences. While the precise mechanisms underpinning increases in extracellular in FW remain poorly understood, findings from *post-mortem* and genetic studies suggest regionally aberrant axonal pruning (Lewis & Levitt, 2002; Mendelsohn, Strous, Bleich, Assaf, & Hendler, 2006; Rapoport, Addington, Frangou, & Psych, 2005), myelin abnormalities (Davis et al., 2003; Uranova et al., 2001), and grey matter loss (Douaud et al., 2007; Höistad, 2009). Higher FW in extracellular tissue may be modulated by neuroinflammatory processes, such as neuroinflammation (Di Biase et al., 2021), atrophy, a lack of dendritic cells, or a breakdown in the cellular membrane (Pasternak et al., 2016; Syková & Nicholson, 2008). It is plausible that the impact of CT is mediated via immune dysregulation: CT is associated with increased levels of inflammatory markers, including Interleukin-6 (IL-6), *tumor necrosis factor-alpha, and* C-reactive protein in healthy participants (Baumeister, Akhtar, Ciufolini, Pariante, & Mondelli, 2016) and stress-induced processes of inflammation have been associated with altered brain structure and function (Corley et al., 2024; Quidé et al., 2020).

No interaction between CT and diagnostic group was detected for FA_T_ or FW, contrary to our hypothesis and inconsistent with reported associations between CT and WM alterations in SZ, particularly in the inferior fronto-occipital fasciculus, the inferior longitudinal fasciculus, and the superior longitudinal fasciculus (Asmal et al., 2019; Domen et al., 2019; Molina et al., 2018; Poletti et al., 2015). By applying a FW correction model to separate the contributions of cellular and extracellular processes, these findings may be more sensitive to detecting microstructural tissue-level changes. Our results are consistent with previous findings that also reported no significant interaction between diagnostic group and CT (Costello et al., 2023). Notwithstanding both sets of findings may be due to a small sample size of 13 participants with high CT levels and/or biases in the recruitment process, which could have favoured more functionally stable individuals with lower exposure to CT and less severe clinical profiles. Additionally, methodological differences relating to the contribution of cellular and extracellular processes cannot entirely explain the lack of a significant association. It is highly probable that the pathways between CT and WM alterations in SZ are complex and highly interrelated (McCarthy-Jones et al., 2018), which is also in keeping with the neural diathesis-stress model (Pruessner, Cullen, Aas, & Walker, 2017; Walker & Diforio, 1997; Walker, Mittal, & Tessner, 2008). Future studies should evaluate more complex and interdisciplinary models, that may also consider altered stress and immune models (Quidé et al., 2020).

We acknowledge several limitations of our study. First, our modest sample size of SZ with high CT levels may have been too small to observe group differences in WM, especially in peripheral WM voxels (Cohens *d* average range = 0.10–0.24) and in correlational analyses which did not include corrections for multiple testing. Future studies with larger sample sizes are needed to confirm the robustness of these findings. Second, history of CT was based on retrospective self-reports, and we did not account for the timing or age of onset of CT, nor the effects of more nuanced aspects of CT, such as witnessing domestic violence, which may have moderated the extent of the WM alterations (Croft et al., 2019). However, the use of the CTQ as the most widely used measurement allows the comparison of findings with other studies. Finally, while comparable results have been obtained using single- and multi-shell data (Bergmann, Henriques, Westin, & Pasternak, 2020), multi-shell diffusion acquisitions may ensure a more robust fit of the FW model (Golub, Neto Henriques, & Gouveia Nunes, 2021).

In conclusion, we demonstrated that both SZ and HC with CT experience showed both higher cellular FA in fronto-thalamic regions and lower extracellular FW. These novel findings suggest that the impact of CT may be independent of SZ, and may reflect a resilience, compensation or adaptation process. Furthermore, these findings may indicate altered inflammatory processes that may be linked to the experience of CT and highlight the need to gain a better understanding of both cellular and extracellular compartments in the diffusion signal. Future work should aim to model the complex interactions of biological and psychosocial mechanisms that may underlie the association between CT and schizophrenia.

## Supporting information

Dauvermann et al. supplementary material Dauvermann et al. supplementary material

## Supplementary material

The supplementary material for this article can be found at https://doi.org/10.1017/S0033291724003064.
