# Supplementary material for: Cellular and extracellular white matter alterations after childhood trauma experience in individuals with schizophrenia
Source: Psychol Med. 2025 Jan 6;54(16):4788–97. doi: 10.1017/S0033291724003064 (PMC11779554; doi:10.1017/S0033291724003064)
Supplement: Dauvermann et al. supplementary material [file S0033291724003064sup001.docx]

**Supplementary Material**

**Cellular and extracellular white matter alterations after childhood trauma experience in individuals with schizophrenia**

**Running title:** Childhood trauma, cellular and extracellular white matter in schizophrenia

Maria R. Dauvermann^a,b**,^, Laura Costello^a,**^, Giulia Tronchin^a^, Emma Corley^a*^, Laurena Holleran^a^, David Mothersill^a,c,d^, Karolina I. Rokita^a^, Ruán Kane^a^, Brian Hallahan^a^, Colm McDonald^a^, Ofer Pasternak^e,f^, Gary Donohoe^a^, Dara M. Cannon^a^

** Joint first authors

**Affiliations**

^a^ Center for Neuroimaging, Cognition and Genomics (NICOG), Clinical Neuroimaging Laboratory, Galway Neuroscience Centre, University of Galway, Ireland, H91TK33 Galway, Ireland

^b^ Institute for Mental Health, School of Psychology, University of Birmingham, B15 2TT, United Kingdom

^c^ Department of Psychology, School of Business, National College of Ireland, Dublin, Ireland

^d^ Department of Psychiatry, Trinity College Dublin, St. James's Hospital, Dublin, Ireland

^e^ Department of Radiology, Brigham and Women’s Hospital, Harvard Medical School, Boston, MA, USA

^f^ Department of Psychiatry, Brigham and Women’s Hospital, Harvard Medical School, Boston, MA, USA

*** Corresponding Author**

Emma Corley

Clinical Neuroimaging Laboratory,

School of Medicine,

University of Galway, Ireland

H91TK33, Ireland.

E-Mail: emma.corley@universityofgalway.ie

**1. Methods**

### 1.1 Participants

### Participants were pooled from a subset of participants recruited as part of the Immune Response & Social Cognition in Schizophrenia project and the Social Cognition study in schizophrenia with identical eligibility criteria, questionnaires and scanning procedures. The Ethics Committees of the following institutions approved both studies: National University of Ireland Galway, University Hospital Galway, and Tallaght Hospital, Dublin. Written informed consent was obtained prior to the initiation of any study-related procedures.

Healthy controls (HC) without a psychiatric condition were recruited from public advertisements placed in County Galway and Dublin. Clinically stable individuals with schizophrenia (SZ) were recruited from hospital outpatient clinics or community-based mental health programs in St. James’s Hospital Dublin or University Hospital Galway via referrals from a trained clinical psychiatrist.

1.2. Procedures

1.2.1. Childhood trauma

Self-reported experiences of trauma during childhood and adolescence were retrospectively assessed using the 28 - item Childhood Trauma Questionnaire (CTQ) (Bernstein et al., 2003). The CTQ is a Likert scale questionnaire that assesses five subtypes of abuse (emotional, physical, and sexual) and/or neglect (emotional and physical) that may have occurred between the ages of 0 - 18 years (Bernstein et al., 2003). The items on each subscale range from ‘1’ (‘never true’) to ‘5’ (‘very often true’). We rescaled the first criterion to reflect the ‘absence’ of scores, and thus, responses ranged from ‘0’ (‘never true’) to ‘4’ (‘very often true’). Therefore, scores summarised across all five subscales provides a total score ranging from ‘0 – 100’ instead of ’25 – 125’, with higher scores indicating more severe traumatic experiences (Bernstein et al., 2003). The validated CTQ-manual cut‐off scores (rescaled) were used to rate the presence and severity of abuse and neglect as either none, low, moderate or severe (Bernstein et al., 2003). Severity for each subscale was calculated individually using the thresholds in the manual and adapted for the rescaled scores: emotional abuse (none 0 - 3; low 4 - 7; moderate 8 - 10; severe 11 - 20), physical abuse (none 0 - 2; low 3 - 4; moderate 5 - 7; severe 8 - 20), sexual abuse (none 0; low 1 - 2; moderate 3 - 7; severe 8 - 20), physical neglect (none 0 - 2; low 3 - 4; moderate 5 - 7; severe 8 - 20), and emotional neglect (none 0 - 4; low 5 - 9; moderate 10 - 12; severe 13 - 20) (Bernstein et al., 2003)**.**

Following this categorization, participants were dichotomized into the groups of either ‘high levels of trauma exposure’ or ‘none-low levels of trauma exposure’. High levels of trauma exposure were defined as the presence of moderate or severe abuse with or without moderate or severe neglect. In contrast, none or low levels of trauma was defined when participants met the criteria for none or low levels across each of the five CTQ subscales or had a single case of moderate or severe neglect (Table 1).

**Supplemental Table 1.** Overview of total CTQ subtype scores for healthy controls and individuals with schizophrenia (n = 166) – Rescaled and original CTQ scores

|  | **Healthy Controls** | **Individuals with schizophrenia** | **Mann-Whitney U** |
| --- | --- | --- | --- |
| **Sample, *n*** | 129 | 37 |  |
| Total CTQ (0-100)  Mean ± SD  Median  Min-Max  Total CTQ (5-125)  Mean ± SD  Median  Min-Max | 11.53 ± 12.02  8  0-52  36.53 ± 12.02  33  25-77 | 18.05 ± 14.6  16  1-72  43.05 ± 14.6  41  26-97 | *U* = 1516.0,  *p =* 0.001***  *U* = 1516.0,  *p =* 0.001*** |
| Emotional abuse  (0-20)  Mean ± SD  Median  Min-Max  Emotional abuse  (5-25)  Mean ± SD  Median  Min-Max | 3.47 ± 4.20  2  0-20  8.49 ± 4.20  7  5-25 | 5.22 ± 4.81  4  0-20  9.70 ± 4.92  9  5-25 | *U* = 1778.0,  *p =* 0.02*  *U* = 2285.0,  *p =* 0.546 |
| Physical abuse (0-20)  Mean ± SD  Median  Min-Max  Physical abuse (5-25)  Mean ± SD  Median  Min-Max | 1.43 ± 2.29  0  0-12  6.43 ± 2.29  5  5-17 | 2.81 ± 4.37  1  0-20  7.19 ± 3.31  6  5-21 | *U* = 1962.0,  *p =* 0.08  *U* = 2130.5,  *p =* 0.286 |
| Sexual abuse (0-20)  Mean ± SD  Median  Min-Max  Sexual abuse (5-25)  Mean ± SD  Median  Min-Max | 0.96 ± 2.95  0  0-18  5.95 ± 2.95  5  5-23 | 1.70 ± 4.44  0  0-18  7.19 ± 5.37  5  5-23 | *U* = 2319.5,  *p =* 0.69  *U* = 2285.0,  *p =* 0.55 |
| Emotional neglect  (0-20)  Mean ± SD  Median  Min-Max  Emotional neglect  (5-25)  Mean ± SD  Median  Min-Max | 1.54 ± 2.64  2  0-18  9.06 ± 4.29  7  5-23 | 3.08 ± 3.39  6  0-20  10.84 ± 4.48  11  5-25 | *U* = 1609.5,  *p* = 0.002*  *U* = 1738.0,  *p* = 0.01* |
| Physical neglect (0-20)  Mean ± SD  Median  Min-Max  Physical neglect (5-25)  Mean ± SD  Median  Min-Max | 0.63 ± 0.94  0  0-3  6.55 ± 2.64  5  5-18 | 0.51 ± 0.93  0  0-3  8.11 ± 3.64  7  5-21 | *U* = 1483.0,  *p =* 0.002***  *U* = 1558.5,  *p =* 0.002*** |

**Abbreviations.** CTQ, Childhood Trauma Questionnaire; SD, standard deviation. (0-100) and (0-20), rescaled scoring system; (5-125) and (5-25), original scoring system.

**Supplemental Table 2.** Childhood trauma severity categorization across each of the five CTQ subscales (n = 166) – Rescaled CTQ scores

| **Childhood trauma severity** | | **Emotional Abuse** | | | | **Physical** **Abuse** | | | | **Sexual Abuse** | | | | **Emotional Neglect** | | | | **Physical Neglect** | | | | |
| --- | --- | --- | --- | --- | --- | --- | --- | --- | --- | --- | --- | --- | --- | --- | --- | --- | --- | --- | --- | --- | --- | --- |
|  | | **None** | **Low** | **Moderate** | **Severe** | **None** | **Low** | **Moderate** | **Severe** | **None** | **Low** | **Moderate** | **Severe** | **None** | **Low** | **Moderate** | **Severe** | **None** | **Low** | **Moderate** | **Severe** |  |
| **None/low**  levels | **HC, *n*** | 78 | 16 | 0 | 0 | 85 | 9 | 0 | 0 | 90 | 4 | 0 | 0 | 73 | 16 | 4 | 1 | 81 | 8 | 5 | 0 |  |
|  | **SZ, *n*** | 16 | 8 | 0 | 0 | 21 | 3 | 0 | 0 | 23 | 1 | 0 | 0 | 11 | 10 | 1 | 2 | 17 | 5 | 1 | 1 |  |
|  | **Total, n** | 94 | 24 | 0 | 0 | 106 | 12 | 0 | 0 | 113 | 5 | 0 | 0 | 84 | 26 | 5 | 3 | 98 | 13 | 6 | 1 |  |
| **High**  levels | **HC, *n*** | 8 | 7 | 10 | 10 | 16 | 7 | 7 | 5 | 18 | 1 | 11 | 5 | 11 | 12 | 5 | 7 | 18 | 7 | 4 | 6 |  |
|  | **SZ, *n*** | 2 | 3 | 3 | 5 | 4 | 3 | 4 | 2 | 7 | 0 | 2 | 4 | 5 | 6 | 1 | 1 | 3 | 3 | 5 | 2 |  |
|  | **Total, *n*** | 10 | 10 | 13 | 15 | 20 | 10 | 11 | 7 | 25 | 1 | 13 | 9 | 16 | 18 | 6 | 8 | 21 | 10 | 9 | 8 |  |

**Abbreviations.** CTQ, Childhood Trauma Questionnaire; HC, healthy controls; SZ, individuals with schizophrenia.

### 1.3 Magnetic Resonance Imaging Data acquisition and processing

Diffusion-weighted MR images (DWI) were acquired for all participants at the Wellcome Trust Health Research Board National Centre for Advanced Medical Imaging (CAMI) at St. James’s Hospital Dublin, Ireland, using a 3.0 Tesla Achieva scanner (Philips Medical Systems, Best, The Netherlands. DWI was acquired using a SE-EPI sequence with a 32-direction Stejskal-Tanner diffusion encoding scheme. Image acquisition parameters were as follows: field of view = 244 x 244 x 140 mm, spatial resolution = 2 mm^3^, 70 slices with no interslice gap, TR/TE = 12807 / 55 ms, SENSE factor = 2, half-scan factor = 0.68, b-values = 0, 1000 s/mm^2^, with SPIR fat suppression and dynamic stabilisation in an acquisition time of 8 minutes and 35 seconds.

All raw DWI were visually inspected prior to tensor-estimation (ExploreDTI v4.8.6). The diffusion-tensor and respective eigenvalues were estimated from DWI using RESTORE (Robust Estimation of Tensors by Outlier Rejection; Chang et al., 2005). Pre-processing was performed to correct for artefacts induced by eddy current distortions, motion, signal dropouts, and susceptibility effects, which also included rotation of the b-matrix (Leemans & Jones, 2009). DWI quality assessment involved manual visual inspection for geometric distortions, large-signal dropouts, abnormal model residuals (Tournier et al., 2011) and registration accuracy which resulted in the removal of ten individuals from all subsequent analyses (four HC and six SZ).

**3. Results**

**Supplemental Table 3.** Significant white matter clusters of reduced tissue specific fractional anisotropy in individuals with schizophrenia group compared to healthy controls

| Cluster | Regions^1^ | Voxels | Max^2^ | Max X (vox)^3^ | Max Y (vox) | Max Y (vox)^4^ |
| --- | --- | --- | --- | --- | --- | --- |
| 1 | - Body of corpus callosum - Superior corona radiata L - Posterior corona radiata L | 1710 | 0.978 | 109 | 99 | 109 |

^1^ Regions identified using the John Hopkins University (JHU) ICBM_DTI_81 White-Matter Labels atlas (Wakana et al., 2007)

^2^ MAX: the value of the z-statistic, 0.95 corresponds to a p-value of 0.05

^3^ MAX X/Y/Z: the location of the maximum intensity voxel, given as X/Y/Z coordinate values in voxel coordinates

**Abbreviations**. L, Left.

**Supplemental Table 4.** Significant white matter clusters of reduced tissue specific fractional anisotropy in the group with high levels of childhood trauma exposure, irrespective of diagnosis

| Cluster | Regions^1^ | Voxels | Max^2^ | Max X (vox)^3^ | Max Y (vox) | Max Y (vox)^4^ |
| --- | --- | --- | --- | --- | --- | --- |
| 1 | - Retrolenticular part of internal capsule R - Posterior corona radiata R - Posterior thalamic radiation (include optic radiation) R - Superior longitudinal fasciculus R | 192 | 0.961 | 58 | 89 | 86 |

^1^ Regions identified using the John Hopkins University (JHU) ICBM_DTI_81 White-Matter Labels atlas (Wakana et al., 2007)

^2^ MAX: the value of the z-statistic, 0.95 corresponds to a p-value of 0.05

^3^ MAX X/Y/Z: the location of the maximum intensity voxel, given as X/Y/Z coordinate values in voxel coordinates

**Abbreviations**. L, Left, R, Right.

**Supplemental Table 5.** Significant white matter clusters of increased free water in individuals with high levels of childhood trauma irrespective of diagnosis

| Cluster | Regions^1^ | Voxels | Max^2^ | Max X (vox)^3^ | Max Y (vox) | Max Y (vox)^4^ |
| --- | --- | --- | --- | --- | --- | --- |
| 1 | - Genu of corpus callosum - Body of corpus callosum - Splenium of corpus callosum - Cerebral peduncle L - Anterior limb of internal capsule L - Posterior limb of internal capsule L - Retrolenticular part of internal capsule L - Anterior corona radiata L - Superior corona radiata L - Posterior corona radiata L - Posterior thalamic radiation (include optic radiation) L - Sagittal stratum (include inferior longitudinal fasciculus and inferior fronto-occipital fasciculus) L - External capsule L - Fornix (cres) / Stria terminalis (cannot be resolved with current resolution) L - Superior longitudinal fasciculus L - Superior fronto-occipital fasciculus (could be a part of anterior internal capsule) L - Uncinate fasciculus L - Tapetum L | 17647 | 0.99 | 118 | 64 | 85 |

^1^ Regions identified using the John Hopkins University (JHU) ICBM_DTI_81 White-Matter Labels atlas (Wakana et al., 2007)

^2^ MAX: the value of the z-statistic, 0.95 corresponds to a p-value of 0.05

^3^ MAX X/Y/Z: the location of the maximum intensity voxel, given as X/Y/Z coordinate values in voxel coordinates

**Abbreviations**. L, Left; R, Right.

**Supplementary Figure 1.** *Significant white matter tissue specific fractional anisotropy of childhood trauma continuous scores, irrespective of diagnosis*

**
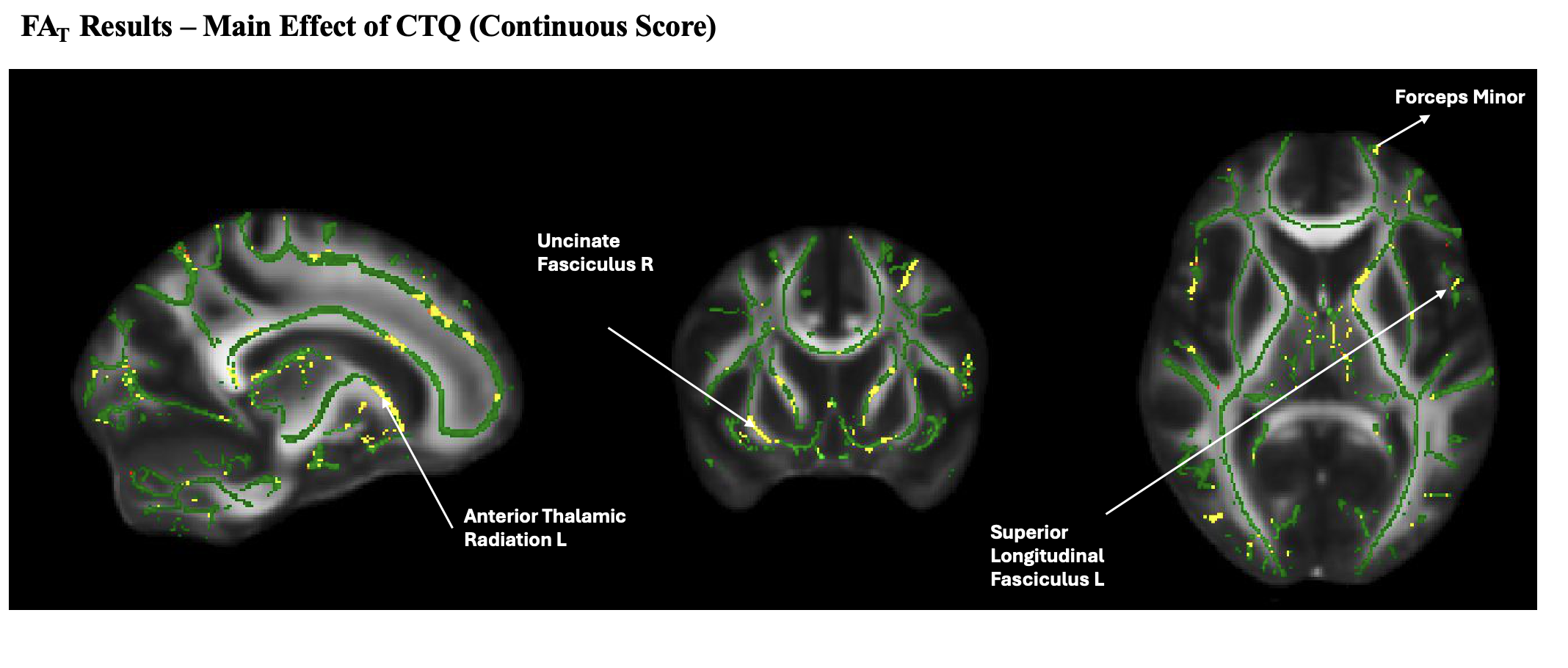
**

**Supplementary Figure 2.** *Significant white matter clusters of increased free water in individuals with higher levels childhood trauma (continuous scores) irrespective of diagnosis*

**
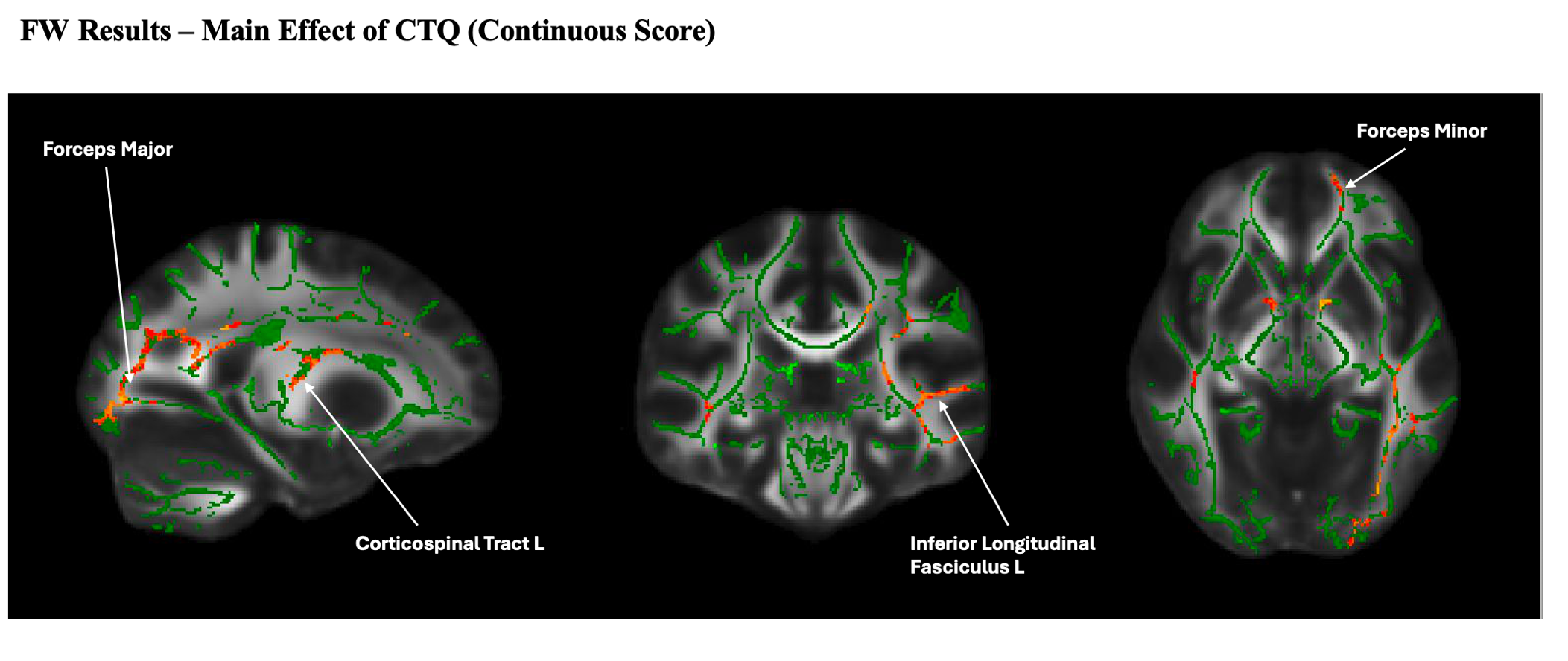
**

**References**

Bernstein, D. P., Stein, J. A., Newcomb, M. D., Walker, E., Pogge, D., Ahluvalia, T., Stokes, J., Handelsman, L., Medrano, M., Desmond, D., & Zule, W. (2003). Development and validation of a brief screening version of the Childhood Trauma Questionnaire. *Child Abuse & Neglect*, *27*(2), 169–190. https://doi.org/10.1016/S0145-2134(02)00541-0

Chang, L.-C., Jones, D. K., & Pierpaoli, C. (2005). RESTORE: Robust estimation of tensors by outlier rejection. *Magnetic Resonance in Medicine*, *53*(5), 1088–1095. https://doi.org/10.1002/mrm.20426

Leemans, A., & Jones, D. K. (2009). The *B* -matrix must be rotated when correcting for subject motion in DTI data. *Magnetic Resonance in Medicine*, *61*(6), 1336–1349. https://doi.org/10.1002/mrm.21890

Tournier, J.-D., Mori, S., & Leemans, A. (2011). Diffusion tensor imaging and beyond: Diffusion Tensor Imaging and Beyond. *Magnetic Resonance in Medicine*, *65*(6), 1532–1556. https://doi.org/10.1002/mrm.22924

Wakana, S., Caprihan, A., Panzenboeck, M. M., Fallon, J. H., Perry, M., Gollub, R. L., Hua, K., Zhang, J., Jiang, H., Dubey, P., Blitz, A., van Zijl, P., & Mori, S. (2007). Reproducibility of quantitative tractography methods applied to cerebral white matter. *NeuroImage*, *36*(3), 630–644. https://doi.org/10.1016/j.neuroimage.2007.02.049
